# Visual capture of gait during redirected walking

**DOI:** 10.1038/s41598-018-36035-6

**Published:** 2018-12-19

**Authors:** Yannick Rothacher, Anh Nguyen, Bigna Lenggenhager, Andreas Kunz, Peter Brugger

**Affiliations:** 1 Department of Neurology, Neuropsychology Unit, University Hospital Zurich, Zurich, Switzerland; 2 Innovation Center Virtual Reality, ETH Zurich, Zurich, Switzerland; 3 Department of Psychology, Cognitive Neuropsychology, University of Zurich, Zurich, Switzerland; 4 Zurich Center for Integrative Human Physiology (ZIHP) and Neuroscience Center Zurich (ZNZ), University of Zurich, Zurich, Switzerland

## Abstract

Redirected walking allows users of virtual reality applications to explore virtual environments larger than the available physical space. This is achieved by manipulating users’ walking trajectories through visual rotation of the virtual surroundings, without users noticing this manipulation. Apart from its applied relevance, redirected walking is an attractive paradigm to investigate human perception and locomotion. An important yet unsolved question concerns individual differences in the ability to detect redirection. Addressing this question, we administered several perceptual-cognitive tasks to healthy participants, whose thresholds of detecting redirection in a virtual environment were also determined. We report relations between individual thresholds and measures of multisensory weighting (visually-assisted postural stability (Romberg quotient), subjective visual vertical (rod-and-frame test) and illusory self-motion (vection)). The performance in the rod-and-frame test, a classical measure of visual dependency regarding postural information, showed the strongest relation to redirection detection thresholds: The higher the visual dependency, the higher the detection threshold. This supports the interpretation of users’ neglect of redirection manipulations as a “visual capture of gait”. We discuss how future interdisciplinary studies, merging the fields of virtual reality and psychology, may help improving virtual reality applications and simultaneously deepen our understanding of how humans process multisensory conflicts during locomotion.

## Introduction

Virtual reality (VR) applications using head mounted displays (HMDs) allow users to deeply immerse in a virtual environment (VE). There is, however, one obstacle to a full-blown immersion, dubbed the “locomotion problem”. It concerns the user’s navigation through large VEs. While virtual worlds can easily be expanded infinitely, physical locomotion remains constrained by the dimensions of the available room. Early attempts to overcome this locomotion problem made use of keyboards and joysticks thus engaging the hands for a task meant, in reality, for the legs. This crude simulation of locomotion was quickly followed by more sophisticated solutions using treadmill-like devices that allow users to physically walk on the spot in order to move through virtual space^1,2^. While such solutions made it possible to “walk” infinitely far in a VE, they are still a cumbersome approximation to real walking. Position tracking technology offers the possibility to translate real walking movements into virtual movements^3^. While this approach produces the exact sensation of real walking, a new problem arises. Due to the HMD, the user is blind to real world obstacles like walls and other objects in the physical room. Because VEs are often larger in size than the available physical space, collisions with physical boundaries become an issue.

**Figure 1:**
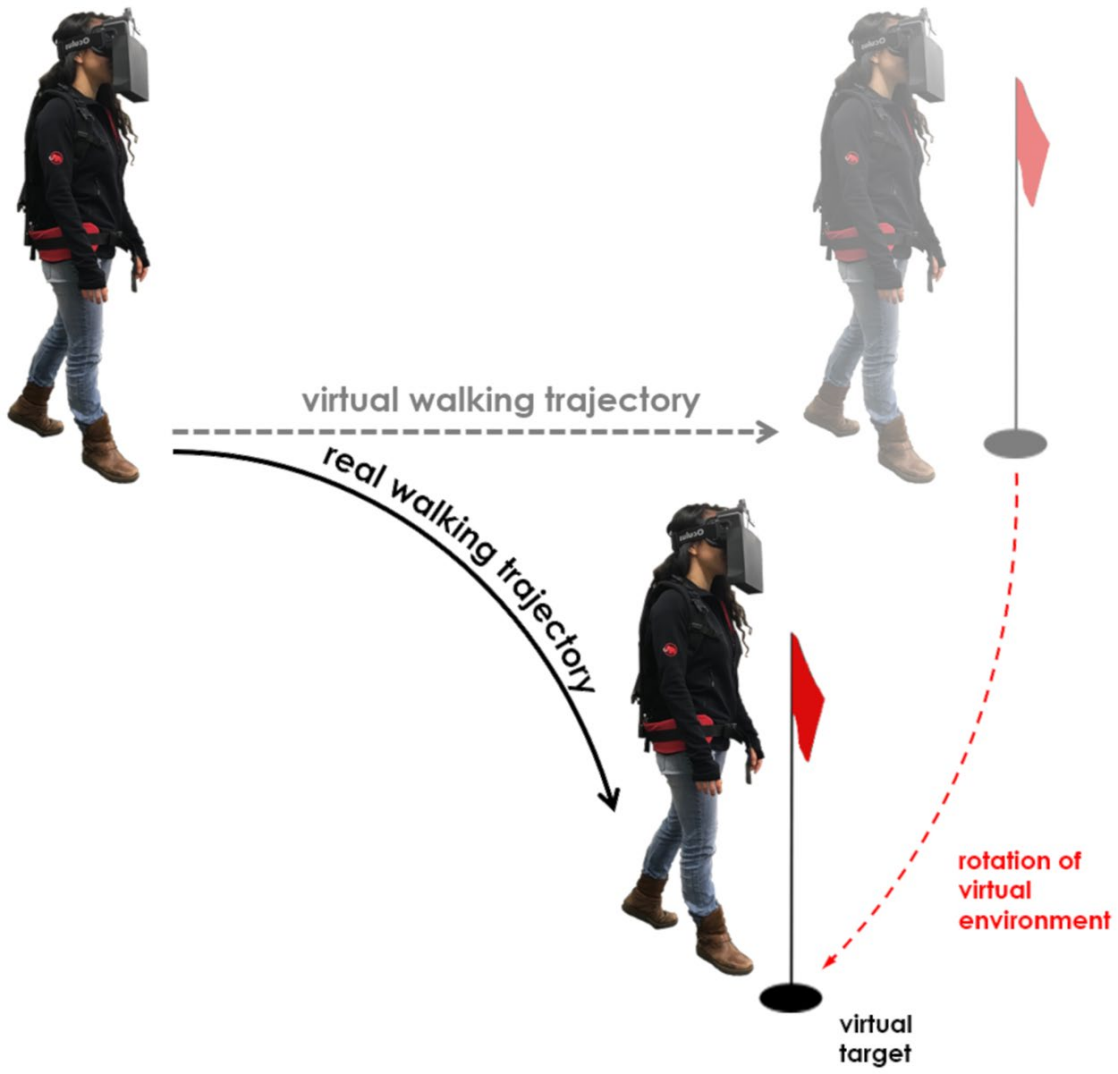
Illustration of a walking trajectory manipulation through redirected walking using a curvature gain.

Redirection or redirected walking is a software-based approach to addressing this problem^4^. By controlling how real-life movements are mapped onto virtual space, redirection aims at manipulating a user’s physical walking trajectory. The focus of the present study is on one key technique of redirection, the so-called “curvature gain”^5^. Applying a curvature gain induces a rotation of the virtual scenery around a user while (s)he is moving. This causes the user to correct for the rotation by walking on a curved pathway (Fig. 1). At the extreme, a user could proceed infinitely far straight forward in the VE while walking in a full circle in reality. Modern algorithms dynamically apply curvature gains based on a user’s position in the virtual and physical environment with the goal to steer the user away from walls, thus making it possible to explore virtual areas much larger than the available physical space^6–8^. The magnitude of a curvature gain is defined as the inverse of the curvature radius.

As long as the radius of a forced curvature is not too small, users fail to notice the manipulation and assume that the virtual walking trajectory corresponds to the real one. Several studies exist that measured the perceptual limits for curvature gains to remain unnoticed. Knowing users’ redirection thresholds is crucial for an immersive virtual reality experience since full immersion will only be guaranteed as long as there is a felt harmony between what one’s eyes see and what one’s legs do. Somewhat surprisingly, the radii of threshold-curvatures vary considerably across several studies, ranging from 5 m to 22 m^9,10^. The variability in these redirection thresholds is possibly due to differences in the applied threshold estimation methods, differences in the architecture and design of the used VEs or more general differences such as dissimilar study populations.

Apart from its value for designing VR-applications, we argue that the issue of redirection is also of considerable interest for psychological research. During walking, redirection causes a mismatch between visual and bodily feedback, the latter comprising vestibular, proprioceptive and somatosensory cues. Experimentally induced sensory mismatch situations have long been used in psychology to study a broad range of phenomena, from motion sickness^11^, multisensory integration^12,13^ and the development of a “bodily self”^14^ to action monitoring^15–18^ and the feeling of agency^19–23^.

More than half a century ago, Nielsen introduced a paradigm, which allowed the manipulation of the visual feedback of a performed hand action, specifically drawing of a straight line in the sagittal direction^24^. With the aid of a mirror, a participant would either see his or her real hand while drawing, or the reflection of the experimenter’s hand in its place. In trials where the experimenter’s hand was visible, the experimenter followed the trajectory of the line produced by the participant but induced a slight lateral deviation. Tricked into believing the experimenter’s hand was theirs, participants, unbeknownst to them, deviated to the opposite side in an attempt to correct for the covert redirection. Nielsen’s paradigm, conceptually reminiscent of redirected walking, proved most influential; it induced a large number of authors to apply modifications of the procedure in the investigation of various aspects of action monitoring and self-recognition. In a seminal study on agency, a sensorimotor adjustment task was applied, in which the visual feedback from goal-directed hand movements was manipulated using angular deviations^18^. This procedure was used to study the conscious monitoring of action in healthy participants. It became again evident that participants automatically adjusted their hand trajectory in order to correct for the induced deviation. Despite this correction, participants ignored the veridical trajectory of their hand when consciously judging its travelled direction. This suggests that participants dominantly relied on visual cues when attributing the observed action, ignoring contradicting proprioceptive signals. Follow-up experiments laid the focus on the cues responsible for the conscious experience of agency. In one exemplary study, participants observed a screen showing either their own hand or the experimenter’s hand^25^. They were required to perform a simple hand motion in response to an acoustic signal while monitoring the visual feedback on the screen. The experimenter would simultaneously perform the same or a different hand motion. Subsequently, participants had to judge whether the observed hand had been their own or the experimenter’s. It could be shown that in the condition where the experimenter performed the same hand movement, the subjects’ performance deteriorated, they mistook the experimenter’s hand as their own in about 30% of trials. It was concluded that subjects based their judgment on slight differences in timing and kinematics, but often these differences were not sufficient to discriminate own from foreign hand actions. These types of paradigms were also applied to clinical populations, classically individuals diagnosed with schizophrenia. Schizophrenic individuals show a disturbance in the attribution of actions, often assigning their own actions and thoughts to alien sources or conversely conceive themselves as the agent of actions performed by others^26,27^. When exposed to the type of experimental situations described above, schizophrenic individuals showed a systematic tendency to attribute the experimenter’s actions to themselves^16,25^.

While initially only motor actions involving the hands were investigated, recent studies have transferred the concept of an induced mismatch between a performed action and its visual feedback to the full body action of walking^23,28^. The participants, who were placed in a tracking area, observed a virtual room projected on a large screen in front of them. An avatar in the virtual room was continuously mimicking the participants’ body-movements. Participants were then required to steer their “virtual body” into a virtual target using real walking. By inducing a lateral drift in the movements of the avatar and testing the participants’ sensitivity to that manipulation, the limits of feeling of agency for human walking were determined^23^. This walking paradigm was recently used in a clinical context and allowed the demonstration that awake sleepwalkers differ from healthy participants in the conscious monitoring of gait^29^.

At this point it becomes evident that although redirected walking has been developed without any intent to apply it in psychological research, the paradigm seems to perfectly line up with the advancement of agency and action recognition studies so far. The above-described setup for studying the feeling of agency in human walking resembles the classical situation of redirection to a remarkable degree. However, some major differences between the two methodologies have to be noted. First, in redirected walking an HMD instead of a projection screen is used, which allows a larger walkable tracking area to be used and avoids any irritation stemming from a mismatch between real and virtual visual cues. Second, redirection is usually applied in a first-person perspective scenario, therefore implementation of a virtual avatar is feasible, but not necessary. This use of a first-person perspective might bring the benefit of a more natural immersion during an experimental procedure. Lastly, the applied manipulations of the walking trajectory differ slightly between the two setups. Common redirection paradigms use a rotation of the VE to influence walking users while traditional work on agency detection have rather introduced a lateral (angular) drift.

Classically, the aim of agency studies based on some modification of the Nielsen paradigm^24^ is to explore the extent to which participants can be disrupted in their feeling of agency. Often this is accompanied by the estimation of a feeling of agency threshold. In the case of redirection, there has also been some effort dedicated to estimating users’ detection thresholds under various conditions^5,9,10,30^. However, the neuropsychological factors determining this threshold have, to the best of our knowledge, never been systematically investigated.

In order to address this gap in knowledge, the present study set out to determine the perceptual and cognitive factors contributing to a person’s ability to detect redirection of gait in a VE. As described above, redirection causes a sensory mismatch between the visual and bodily feedback during walking. Previous studies on action recognition and basic research in multisensory processing would suggest that visual dominance over the other senses is responsible for the incomplete awareness of being redirected along a visually guided path. In situations of multisensory conflicts, human observers tend to believe their eyes rather than their ears^31^, their proprioceptive sense^32^, or their vestibular perception^33^. This dominance effect is generally interpreted as a “visual capture” of non-visual senses. Alternatively (or in addition), studies investigating locomotor control in blindfolded walkers have proposed a general insensitivity to noise in the motor output, and thus a lack of non-visual body awareness, as a key factor limiting healthy subjects’ sensitivity to detect curved walking trajectories^34,35^.

**Figure 2:**
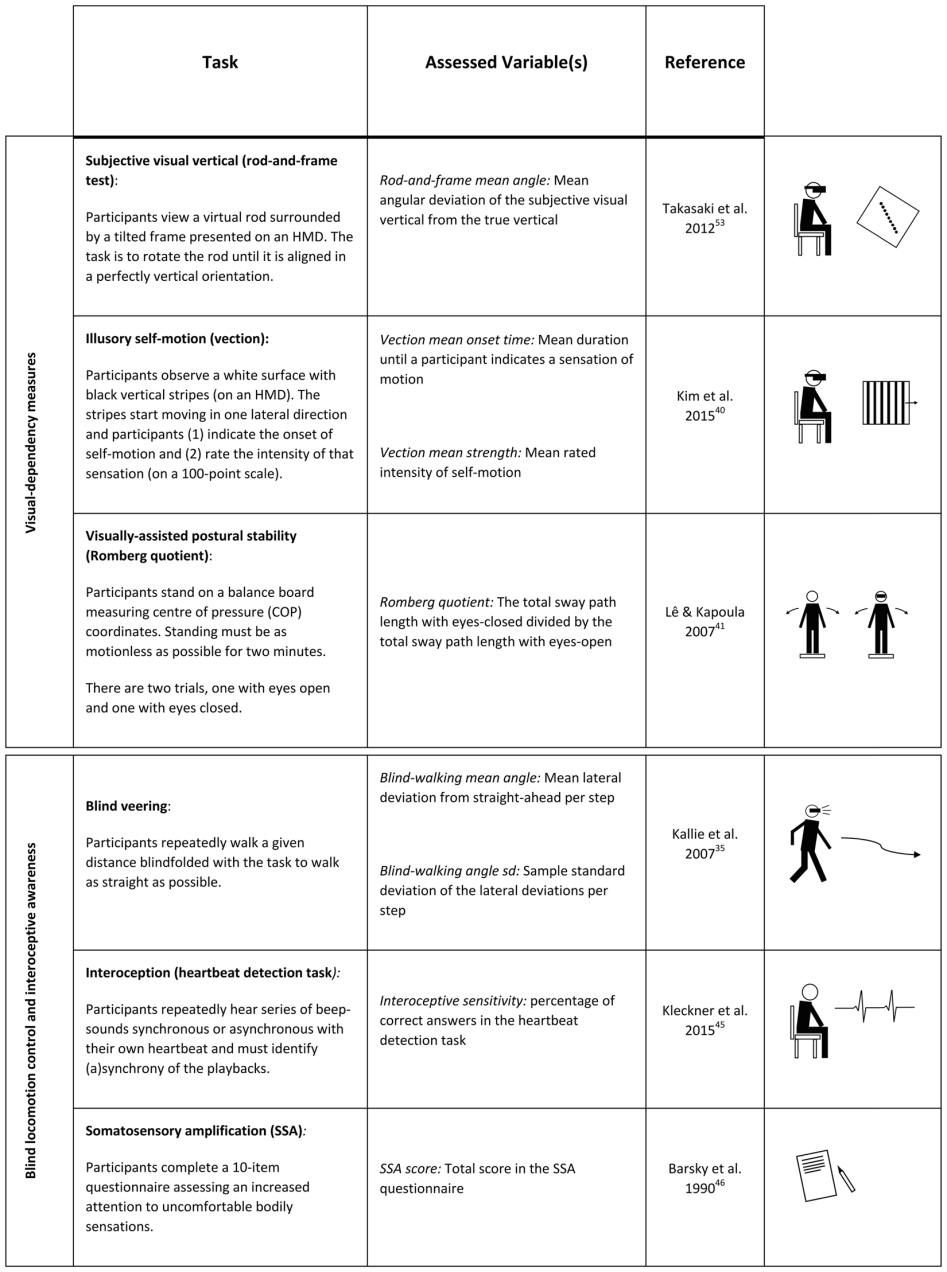
Short descriptions of the applied tasks and the derived variables. For more details see text.

To test whether similar mechanisms are responsible for the perception of redirected walking, we assessed healthy participants’ curvature gain detection thresholds in a well-defined redirection paradigm and had each participant perform a series of carefully selected tasks. In total, six different tasks were employed with the goal to capture the full range of psycho-physical traits suspected to be of importance for the detection of redirection (see Fig. 2). The six applied tasks were divided into two groups, where the first group addressed visual dependency measures (i.e. the degree of reliance on visual cues relative to cues of other modalities; Fig. 2, top), while the second group assessed non-visual body control and awareness (Fig. 2, bottom).

Given the wide variety of cues and modalities, in relation to which visual dependency can be examined, the first three tasks were selected with the intent to measure visual dependency specifically regarding gait-relevant sensory cues. As a result, the three visual dependency measures included 1) visual dependency in relation to postural information using a rod-and-frame test, 2) visual dependency in relation to experience of self-motion using a vection susceptibility test, and 3) visual dependency in relation to postural control using a Romberg test.

The rod-and-frame test, originally developed by Witkin and Asch in the late 1940s^36^, requires a subject to align a rod inside a slanted frame until it is perceived as vertical. Originally devised to assess human subjects’ “sense of space”^36^, the rod-and-frame test has advanced to a standard procedure to study interactions between the visual, vestibular and proprioceptive senses^37^. Today the test is commonly used as a visual dependency measurement and assesses the degree to which a person relies on visual rather than postural information to judge the gravitational vertical. Interestingly, and perhaps even more relevant for the case of redirection, performance in the rod-and-frame test has also been linked to body-awareness, showing that subjects with lower rod-and-frame test performance (“field-dependent”) are more susceptible to the rubber hand illusion^38^. In order to perform well in the rod-and-frame test, the tested person has to focus on vestibular and proprioceptive cues while ignoring the visual experience of the slanted frame. We hypothesized that during redirection, participants also must focus on postural information while ignoring contradicting visual cues to detect the curved walking trajectory. Thus, we expected participants, who perform better at the rod-and-frame test, to be less easily tricked by redirected walking.

Vection designates the illusory perception of own-body movement during the visual observation of moving objects in the environment and is commonly used as a test of visual dependency in visual-vestibular conflicts^39,40^. Vection is the powerful sensation known by everybody who has ever looked out through the window of a stationary train, thinking that it is moving just before noticing that in fact it is the train on the opposite track that has started to move. Specifically, *circular* vection refers to the illusion of self-rotation on observing vertical stripes on a surrounding surface (traditionally an “optokinetic drum”) that moves around the upright axis of the body. Similar to circular vection, redirected walking exposes participants to a visual-vestibular conflict due to the visual rotation of the VE around the moving participant. We hypothesized that participants, who are more visually dominated in visual-vestibular conflicts and thus more susceptible to vection, would also have more difficulties detecting the visual-vestibular mismatch elicited by the rotating VE during redirected walking.

The Romberg test is a standard tool used in sway analysis and aims at identifying the influence of vision on postural control^41^. The amount of body sway occurring when attempting to stand still reflects a person’s postural stability. Therefore, visual dependency in relation to postural control can be measured by how much the body sways when the eyes are opened compared to when the eyes are closed. The ratio of the sway path lengths under these two conditions is referred to as the Romberg quotient. Similar to the rationale behind the rod-and-frame test and the vection susceptibility test we hypothesized that participants who are less visual dependent in their postural control would have less difficulties detecting disruptions in their postural balance due to the curved walking trajectory, despite contradicting visual inputs.

In addition to these three tasks assessing different facets of visual dependency, three further tasks were chosen to assess non-visual body control and awareness. These tasks aimed at measuring 1) participants’ non-visual locomotor control using a blind veering test, 2) participants’ sensitivity to interoceptive cues using a heartbeat detection task, and 3) participants’ sensitivity to somatosensory inputs using the somatosensory amplification (SSA) scale.

Blind veering refers to any lateral deviation when trying to walk a straight line without any visual feedback^35^. With the visual feedback completely removed, the blind veering test assesses a person’s ability to identify a straight walking direction using only non-visual cues. Therefore, the performance in a blind veering test is a measure of a person’s bodily awareness. Since the detection of redirected walking requires participants to identify non-straight walking trajectories, we hypothesized that a heightened awareness of non-visual locomotor-related cues, as measured by the blind veering test, would serve as an advantage in detecting redirection.

In contrast to the highly locomotion-specific form of body awareness assessed by the blind veering test, a more generic type of body awareness can be measured using interoception tests. Interoception describes the perception of the internal state of the body and its visceral organs^42^. The importance of interoception for navigating in VEs has recently been emphasized^43^. Also, a high interoceptive sensitivity has previously been linked to a lowered propensity for experiencing a bodily illusion resting on multisensory conflict^44^. Classically, interoception is measured in cardiac-based tasks. The heartbeat detection task requires a person to identify whether a series of acoustic signals is synchronous or out-of-sync with his/her heartbeat^45^. A person, who performs well in this test, could be assumed to be more aware of their internal cues, potentially being less distracted by concurrent visual cues. We thus hypothesized that a generic bodily awareness, expressed by a person’s interoceptive sensitivity, would facilitate the detection of the body’s curved walking trajectory during redirection.

Finally, the SSA scale questionnaire can be used to assess a heightened attention to uncomfortable bodily sensations^46^. We hypothesized that such a heightened attention could be beneficial in detecting potential, uncomfortable bodily sensations triggered by redirection.

## Methods

### Participants

Sixty persons, 30 women and 30 men, aged between 18–35 years (mean age: 25.1 years, sd: 3.9 years) participated in the study. Their average duration of education was 16.1 years (sd: 3.8 years), and on average they spent 1.8 hours a week video gaming (sd: 3.9 hours) and 3.4 hours a week exercising (sd: 3.2 hours). Exclusion criteria included any history of neurological or vestibular disease and any type of injury affecting natural walking. Participants were right-handed according to a validated lateral preference inventory^47^. They were mostly recruited through the online market-place of the University of Zurich. The participants signed an informed consent sheet prior to starting the experiment. All experimental procedures were approved by the Cantonal Ethics Committee of Zurich (BASEC Number: 2016-01153) and carried out in accordance with the ethical standards of the Declaration of Helsinki.

### Redirection procedure and threshold assessment in a VE

**Figure 3:**
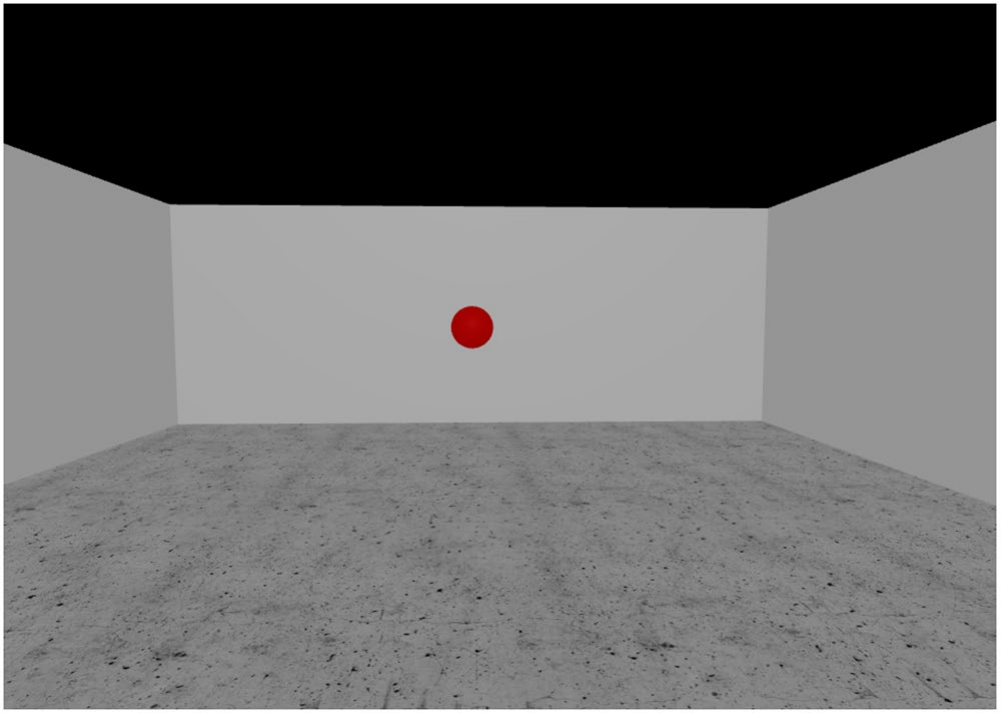
Screenshot of the virtual environment used in the redirection threshold assessment. The red sphere, serving as the participants’ target, is visible in the middle.

Participants wore an Oculus DK2 HMD and were connected to an Intersense IS-1200 optical tracking system for 6 DOF head position tracking at 180 Hz^48^. They started at one end of a 12 m × 6 m tracking area and found themselves in an empty virtual room with a floating red sphere 7.5 m in front of them (Fig. 3). Redirection thresholds were determined in a two-alternative forced choice task (2AFC task). Participants were asked to walk straight to the virtual target (floating red sphere) for two consecutive trials. Just in one of these two trials, a curvature gain of a predefined intensity was applied. The task for the participants consisted of identifying, immediately after the completed two trials, in which trial the redirection had taken place. An eye-tracker integrated in the HMD allowed the participants to give their answer by looking at corresponding icons (“first trial” vs. “second trial”) shown on the display. Depending on whether the answer was correct or not, the intensity of the curvature gain was automatically adapted for the next round in order to make the task more or less difficult. Whether redirection was applied in the first or second trial was randomly distributed. We used the Bayesian-based adaptive method QUEST to choose appropriate stimulus levels for each round and to estimate the final threshold value^49^. The performance in such a 2AFC task is modelled as a psychometric curve, in which the probability of a correct answer is plotted against the applied stimulus intensity (in our case curvature gain magnitude). The psychometric curve theoretically starts at a guessing rate of 50% for low stimulus levels and approaches a 100% detection rate with increasing stimulus intensity. The detection threshold on this curve is classically defined as the stimulus level with a 75% detection rate. We used separate, interleaved QUESTs for each participant in order to estimate independent detection thresholds for left- and right-ward redirection. In total, each participant completed 160 rounds, each consisting of two trials. Walking speed was controlled by a metronome, to which the participants were asked to adjust their step frequency. The frequency of the metronome was adapted to each participant based on the formula by Dean, 1965^50^, targeting an average walking speed of 1 m/s. Before starting the threshold assessment procedure, participants completed a set of practice trials to make sure that the task was understood, and that the adaptation of the walking speed and the use of the eye-tracker were sufficiently mastered. A custom-made cover in front of the HMD ensured that participants could not get any visual cues from their surroundings.

### Perceptual and cognitive tasks

Figure 2 provides an overview of the tasks and variables used to determine the factors related to participants’ redirection thresholds. In total, participants completed the following six tasks: (1) A rod-and-frame test, which is the classical measurement of visual dependency regarding vestibular and proprioceptive signals; (2) a vection susceptibility test, which is an assessment of visual dependency during judgements of illusory self-motion; (3) a Romberg test, which determines the visual dependency in postural stability; (4) a blind veering task, which quantifies non-visual, locomotion-related body control; (5) a heartbeat detection task, which scores the participant’s interoceptive abilities; and (6) a somatosensory amplification questionnaire, which assesses an increased attention to uncomfortable bodily sensations.

The procedures of the single tasks are introduced here in more detail and grouped according to whether they involve the visual sense or rather emphasize non-visual, locomotion-related processing or interoceptive awareness.

#### Tasks mainly involving vision

Subjective visual vertical (rod-and-frame test): For our study we deployed a VR-adapted version of the rod-and-frame test^51^. Participants wore an Oculus DK2 HMD while sitting upright on a chair. On the HMD, they were presented a virtual, tilted frame surrounding a rod that could be rotated using a joystick. They were required to set the rod in a perfectly vertical orientation. The virtual rod was composed of a dotted line to prevent giving any cues about its orientation due to the limited resolution of the HMD^52^. Each participant completed 20 trials consisting of a randomized, balanced set of a frame tilted ± 20° paired with a rod, initially tilted ±18° (after Takasaki *et al*., 2012^53^).

Based on the 20 trials, the *rod-and-frame mean angle* was calculated for each participant. This is the average of the 20 (unsigned) angular deviations of the subjective visual vertical from the true vertical.

Illusory self-motion (Vection): For the vection susceptibility test, participants were seated upright on a stationary chair and observed a pattern of vertical black and white stripes on an Oculus DK2 HMD. Upon button press, the virtual drum started rotating, increasing rotation speed for 6 seconds (acceleration: 10 degree/s^2^) and ending up at a constant speed of 60 degree/s (based on Melcher & Henn, 1981^54^). Participants had to focus on the moving surface and report by button press as soon as they felt the sensation of themselves rotating instead of merely looking at a rotating surface. If no such sensation occurred, the simulation would stop after 30 seconds. Following each trial, participants had to rate the strength of the movement sensation on a scale of 0–100, with 0 denoting no sensation of movement, and 100 denoting a sensation indistinguishable from real movement. In total, participants completed eight trials, consisting of four left-ward and four right-ward rotations presented in a randomized order.

Based on these eight trials, the *vection mean onset time*, which is the average duration until a participant presses the button indicating a sensation of movement, and the *vection mean strength*, which is a participant’s average rating of the strength of his/her sensations of movement, were calculated for each participant.

Visually-assisted postural stability (Romberg quotient): The amount of body sway occurring when attempting to stand still reflects a person’s ability of postural control. Using a Wii balance board^55^, each participant was instructed to stand as still as possible without shoes in a natural, shoulder wide stance, arms hanging down on the side under two conditions. In the first condition, participants kept their eyes open while fixating a cross at eye level 40 cm in front of them. In the second condition, participants kept their eyes closed. Each condition lasted two minutes. During both conditions, the coordinates of the participants’ centre of pressure (COP) were recorded.

Using the recorded COP coordinates, the total sway path length was calculated for the eyes-open and eyes-closed condition. In order to quantify the influence of vision on sway, the *Romberg quotient* was calculated for each participant. This is the common measure of visual dependency in sway analysis^41^. The *Romberg quotient* is derived by dividing the total sway path length with eyes closed by the total sway path length with eyes open.

#### Tasks focusing on non-visual senses

Blind veering: To quantify a person’s blind veering tendency, we adapted the procedure described by Kallie *et al*., 2007^35^. The same VR set up used for the redirection threshold estimation was employed. Participants wore an Oculus DK2 HMD and were connected to an Intersense IS-1200 optical tracking system for 6 DOF head position tracking at 180 Hz^48^. The participants started at one end of the 12 m × 6 m tracking space and were exposed to a completely dark VE except for a floating red sphere 9.5 m in front of them. Participants were instructed to walk straight towards this sphere. After a walked distance of 1 m, the red sphere disappeared, leaving the moving participants effectively blindfolded. Participants were beforehand instructed to keep on walking as straight as possible, as if the target were still visible. In each trial participants were required to walk until they crossed the frontoparallel plane located 8 m from the starting position, at which point an instruction to stop appeared on the HMD. In total, each participant completed 40 trials. A cover in front of the HMD ensured that participants could not get any visual cues from their surroundings. Walking speed was controlled by a metronome, to which the participants were asked to adjust their step frequency. The frequency of the metronome was adapted to each participant based on the formula by Dean, 1965^50^, targeting an average walking speed of 0.75 m/s. Based on the head tracking data, the walking trajectories of the participants were generated and separated into individual step-vectors (using oscillatory head-movements to recognize single steps).

Based on the step-vectors, the *blind-walking mean angle* was calculated for each participant, which is the average (unsigned) direction deviation per step related to the preceding step in degrees, thus reflecting how straight a participant walked on average. Additionally, the *blind-walking angle sd* was calculated for each participant, which is the sample standard deviation of the step deviations, a measure that has previously been reported to be related to curvature sensitivity^35^.

Interoception (Heartbeat detection task): Quantifying interoceptive sensitivity is most commonly done in tasks, during which participants are asked to track or detect their own heartbeats. The heartbeat detection task deployed here generally follows the procedures proposed by Kleckner *et al*., 2015^45^. Participants were connected to a three-electrode electrocardiogram (ECG) using an e-health sensor platform^56^. The participants were asked to feel their pulse on the wrist and were presented with the two conditions of the heartbeat detection task. In the synchronous condition, an acoustic signal is played 200 ms after an R-spike in the ECG, which has been shown to be perceived as simultaneous with the felt heartbeat in the body^57^. In the asynchronous condition, a delay of 500 ms is added between an R-spike in the ECG and the playback of the acoustic signal, which has been shown to be perceived as not synchronous with the felt heartbeat^57^. After participants had experienced the difference between the two conditions while feeling their pulse, the heartbeat detection task began. While sitting upright on a chair without leaning on the backrest and with the hands placed on the legs (palms facing upwards) the participants were presented with 10 seconds of either a synchronous or asynchronous playback. Participants were instructed to feel their heartbeat and to identify whether a synchronous or asynchronous playback had been presented. After the verbal response was given to the experimenter, the next trial was initiated. In total, each participant completed 40 trials balanced for synchronous and asynchronous trials (in randomized order), with a short (approx. 2 minutes) break after 20 trials. The heartbeat detection task was created and presented using the software ExpyVR (http://lnco.epfl.ch/expyvr).

Each participant’s *interoceptive sensitivity* was calculated, which is the percentage of correct responses in the heartbeat detection task.

Somatosensory amplification: Participants completed the SSA scale questionnaire, which has 10 items scored from 1 to 5^46^. For each participant, the *SSA score* was calculated as the sum of the scores for the single items.

### Statistical analysis

Outlier removal was conducted for all tasks based on the median absolute deviation. Data points in each variable were classified as outliers if they were located further from the median than three times the median absolute deviation, an approach considered a very conservative outlier detection measure^58^. This outlier removal procedure resulted in the loss of seven data points of a total of 476 data points (in four participants the interoception task could not be performed because no reliable heartbeat signal could be obtained). To inspect the correlations among the included variables, a correlation matrix was created using Pearson correlation coefficients.

Before examining the performances in the perceptual-cognitive tasks, we made a univariate assessment of the effects on redirection thresholds of gender, curvature direction, years of education, weekly gaming hours and weekly sports hours. These potential confounders were then combined into a basic linear mixed model, which included participant ID as a random intercept and redirection threshold as the target variable.

We tested each of the eight perceptual-cognitive task variables for a possible relation with redirection thresholds by adding them to this basic model without any of the other perceptual-cognitive variables (linear mixed model: redirection threshold ~ gender + curvature direction + education + sport/week + gaming/week + (1 | participant) + *tested variable*). These models for an individual assessment of the perceptual-cognitive variables are from here on still referred to as “univariate models”, although the above listed potential confounders remained included. Eventually a final model was fitted (from here on referred to as the “multivariate model”) using a forced-entry approach, meaning all factors and variables were included. In order to compare the effects between variables, standardized coefficients were calculated for the multivariate model. Confidence intervals based on parametric bootstrapping were computed for all coefficients.

Residual analysis of the used linear mixed models was performed by inspecting the associated Tukey-Anscombe plots and the Q-Q-plots of the random factor and the residual error. No transformations of the data were applied. All statistical tests were performed using the software R^59^ with a significance level of α = 0.05 (statistical significance was always assessed in a 2-sided fashion). Fitting of the mixed linear models and testing of the coefficients was conducted using the “lme4” and “lmerTest” package^60^.

## Results

Descriptive statistics, the final number of data-points per variable, and the correlations between the included variables are presented in Table 1.

**Table 1:** Correlation matrix of all tested variables, listing descriptive statistics (sample size, mean, standard error of the mean) and the Pearson’s correlation coefficients.

		N	Mean (s.e.m.)	1	2	3	4	5	6	7	8
1	*Rod-and-frame mean angle*	58	4.78° (0.34)								
2	*Vection mean onset time*	60	17.79 s (0.83)	0.05							
3	*Vection mean strength*	60	39.29 (2.89)	0.28*	−0.45***						
4	*Romberg quotient*	58	1.19 (0.02)	−0.22	−0.22	0.05					
5	*Blind-walking mean angle*	58	0.58° (0.06)	0.22	−0.21	0.15	−0.05				
6	*Blind-walking angle sd*	59	1.89° (0.05)	−0.11	−0.16	0.01	−0.08	−0.13			
7	*Interoceptive sensitivity*	56	0.55 (0.01)	−0.10	0.08	−0.05	0.04	−0.05	0.05		
8	*Somatos. amplif. score*	60	30.72 (0.59)	0.05	−0.24	0.22	−0.05	0.19	0.18	0.15	

Statistical significance (two-sided) is represented as: *p < 0.05, **p < 0.01, ***p < 0.001.

The participants’ mean redirection threshold (taking the average of the left- and rightward curvature threshold per person) is a curvature gain of 0.105, which corresponds to a curvature radius of 9.55 m (95%CI: 8.78 m−10.48 m).

The results from the multivariate and the univariate models, assessing the relation of redirection thresholds with the perceptual-cognitive task performances, are listed in Table 2. In the univariate assessment, gender showed a significant effect (β  = −0.022, p = 0.016), with men having lower redirection thresholds than women. None of the other potential confounders showed any significant effect. Of the perceptual-cognitive variables, the *rod-and-frame mean angle* (β  = 0.006, p = 0.002), the *Romberg quotient* (β = −0.078, p = 0.017) and *vection mean onset time* (β  = 0.002, p = 0.037) showed significant effects in the univariate models. In the multivariate model, the *rod-and-frame mean angle* (std. β  = 0.324, p = 0.019) remained to be a significant predictor of redirection thresholds. Of all included variables in the multivariate model, the *rod-and-frame mean angle* showed the largest standardized coefficient.

**Table 2:** Results from the univariate models and the multivariate model, testing the relations of the assessed variables with redirection thresholds.

	Univariate			Multivariate		
	Coefficient	P-value	95% CI	Stand. coefficient	P-value	95% CI
*Gender (male)*	−0.022	0.016*	−0.040 −0.004	−0.41	0.104	−0.905 0.134
*Curvature direction (right)*	0.005	0.344	−0.006 0.016	0.17	0.199	−0.092 0.424
*Education*	0.002	0.124	−0.001 0.004	−0.10	0.497	−0.379 0.200
*Sport per week*	−0.002	0.145	−0.005 0.001	−0.12	0.321	−0.350 0.117
*Video-gaming per week*	0.000	0.816	−0.002 0.003	−0.00	0.990	−0.289 0.321
**Perceptual-cognitive variables**						
*Rod-and-frame mean angle*	0.006	0.002**	0.002 0.010	0.32	0.019*	0.066 0.611
*Vection mean onset time*	0.002	0.037*	0.000 0.003	0.20	0.164	−0.110 0.472
*Vection mean strength*	0.000	0.513	−0.000 0.001	0.17	0.190	−0.078 0.410
*Romberg quotient*	−0.078	0.017*	−0.138 −0.017	−0.16	0.202	−0.421 0.073
*Blind-walking mean angle*	−0.009	0.344	−0.029 0.009	−0.08	0.479	−0.334 0.140
*Blind-walking angle sd*	0.011	0.312	−0.012 0.033	0.11	0.377	−0.148 0.360
*Interoceptive sensitivity*	0.033	0.536	−0.078 0.126	0.05	0.635	−0.176 0.317
*SSA score*	−0.002	0.089	−0.004 0.000	−0.20	0.122	−0.412 0.035

Statistical significance (two-sided) is represented as: *p < 0.05, **p < 0.01, ***p < 0.001.

**Figure 4:**
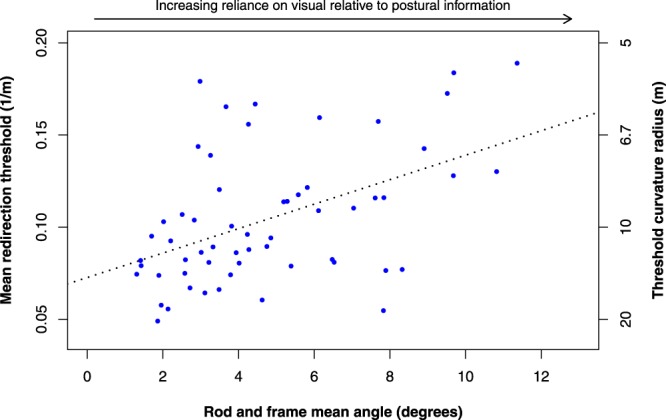
Mean redirection thresholds plotted against the variable *rod-and-frame mean angle*. Mean redirection thresholds are given in gain units and the corresponding curvature radii. The regression line is shown (R^2^ = 0.23, p = 0.0002).

Figure 4 shows the relationship between individuals’ redirection thresholds (taking the average of the left- and rightward curvature threshold per person) and the *rod-and-frame mean angle*.

## Discussion

We set out to determine participants’ redirection detection thresholds and the factors of the visual-vestibular (and proprioceptive) integration process that best predict these thresholds. To this end we assessed in 60 healthy volunteers, (1) their redirection thresholds in a VE, and (2) their performances in a series of tasks that quantify the reliance on visual cues during visual-vestibular conflicts and the non-visual monitoring of locomotion, balance and one’s internal bodily state.

On average, participants noticed a curvature radius of 9.55 m. This finding lines up well with existing results from previous redirection threshold studies, in which curvature radii between 5 m and 22 m were reported^9,10^. Other studies that also introduced multisensory conflicts during walking, particularly in the field of agency, reported thresholds in terms of angular drifts instead of curvature radii^23^. Although there are differences between the manipulation methods used in redirection and agency studies, as outlined in the Introduction section, a conversion of an angular drift to a curvature gain is conceivable. Under the assumption that users correct for an angular drift by walking on a curved pathway, initially facing the virtual target, the radius of that curvature and thus the corresponding curvature gain can be calculated. Following this conversion, the reported agency thresholds for human walking of 10 to 15 degrees angular deviation over a distance of 1.5 m^23^, can be expressed as curvature gains of 0.24 to 0.36. Interestingly, this gain range seems to be rather high compared to the threshold gain found in our study. This would suggest that a stronger manipulation is needed to lose the feeling of agency over walking compared to detecting redirection, which agrees with existing findings from hand-based visuomotor incongruency tasks^61,62^.

Regarding the cognitive and perceptual tasks, our univariate analyses revealed that individual redirection thresholds were related to the performance in three tasks measuring the subjective visual vertical, illusory self-motion while observing optic flow and the extent to which balance can be maintained in a visually-guided condition relative to a blind condition. We proceed to discuss these findings in turn.

### Subjective visual vertical

We used the rod-and-frame test as the classical procedure to measure visual dependency in adjusting the subjective visual vertical. The extent, to which a sitting individual’s perception of verticality is influenced by a visually presented distractor frame, is taken as an indicator of his or her reliance on visual relative to postural cues. We found this relative reliance to be associated with participants’ redirection thresholds in that the less visually dependent a person is in estimating the subjective visual vertical, the less easily he or she was tricked by the redirection manipulation. For the neuroscientific study of gait and agency, this indicates that the interplay between postural and visual processing even outside the context of locomotion can be taken as a proxy to investigate the complex control of walking, both covert and overt^23^. For the VR community, a practical consequence of this finding could be that newly developed techniques of redirection should be evaluated in light of participants’ performance in a brief but standardized assessment of the subjective visual vertical.

We found a gender effect in the rod-and-frame test performance, consistent with numerous previous reports^63,64^. Men were more accurate (i.e. less field-dependent) than women (Wilcoxon rank sum test: W = 274, p = 0.022). It may thus not be a surprise that, as a group, men also showed lower redirection thresholds in the VR setting, a finding to our knowledge not previously reported in literature. Men’s superior performance in both the adjustment of the visual vertical and the judgment of presence/absence of an imposed locomotor perturbation can theoretically be accounted for by two different mechanisms. Either it is a consequence of their generally better spatial abilities^65^ or it is due to sex differences in graviception^66,67^, which may themselves rest on the anatomy of the otolithic system reportedly different in women and men^68^. Whatever the ultimate reasons are for the gender effects found in the present study, they may indicate that the gender differences in the use of VR will remain a tangible reality (https://www.huffingtonpost.co.uk/ailsa-wakley/women-in-vr_b_15403772.html, last accessed November 18, 2018).

### Illusory self-motion

Vection susceptibility has long been known as a measure of visual dependency in visual-vestibular interactions^40^. We assumed that those participants with high vection susceptibility (i.e. participants with small vection onset times and/or high vection vividness) would show a poor awareness of any imposed redirection while walking. However, this assumption was not confirmed by our study. On the contrary, our experiment produced significant results in the opposite direction: Participants with smaller vection onset times, who accordingly were experiencing the illusion of vection faster, were performing *better* at detecting redirection in the VE. Maybe our assumption that these participants would also be those being more dependent on visual cues was not justified. Note that there was no significant correlation between visual dependency as established by the rod-and-frame test and the vection onset-time. The absence of a correlation between the two measures might be explained by the different natures of the rod-and-frame task and the vection measurement. While the rod-and-frame task assesses visual dependency specifically in relation to postural information under rather static conditions, the vection test examines visual dependency in relation to perception of self-motion using more dynamic visual stimuli. We also note that the subjectivity of vection measurement is a frequently cited methodological drawback, and more objective measures of vection susceptibility are still missing^69^. Perhaps participants with short vection onset times were simply better in experiencing the illusion because of a more rapid visual-vestibular integration. In the VR setting, this integration advantage may have led to a better detection of the redirection manipulation.

We emphasize that illusions of self-motion are not as easily elicited in an HMD, than under conditions of full-field optic flow observed in an optokinetic drum^40^, due to its smaller field of view. This may have prevented us from uncovering gender effects reported occasionally for non-computerized tests of vection^70^. In fact, a recent study on circular vection induced by optic flow on an HMD has likewise described an absence of gender effects^71^.

### Visually-assisted postural stability

Standing as still as possible does not guarantee absence of measurable body sway. We focused on this kind of unintentional sway under two conditions: Blind versus fixating on a visual target. The *Romberg quotient* (ratio between sway without and sway with visual control) is a measure routinely used in neurological assessments of postural stability^41^. Again, we expected participants who are less visually dependent in balance control, i.e. whose Romberg quotient is close to 1, to be more sensitive to being redirected while walking toward a visual target. And again, like in the case of vection, our findings are opposite to this prediction. The more a participant profited from visual guidance, the *lower* was his or her redirection threshold. In order to elucidate the counterintuitive direction of the observed relationship, we correlated individual Romberg quotients and the sway path length observed under eyes-closed and under eyes-open conditions. We found that the Romberg quotient was highly correlated with the total sway path length in the former condition (R = 0.57, p = 2.4 * 10^−6^) and weaker with the sway path length in the latter (R = − 0.34, p = 0.008). This raises doubt in traditional interpretations of the Romberg quotient as primarily reflecting the ability to dampen sway by visual information. It rather suggests that the size of the quotient is a more direct function of body sway in the absence of vision. Blind sway also showed a significant negative relation with redirection thresholds when tested univariately (β = −0.0004, p = 0.038), but no significant effect was found for visually guided sway ((β = 0.000, p = 0.96). This indicates that a lower postural balance stability is associated with a better ability to detect redirection. One possible explanation for this association is that participants with relatively poor balance control are also those, who are more easily thrown out of balance by even small curvature gains. Even if only post-hoc, this interpretation should be tested in future work by assessing body sway during locomotion in a VE with various forms of redirection.

Previous studies have reported correlations between visual dependency in the rod-and-frame test and postural stability. Specifically, reliance on visual cues in the rod-and-frame test were associated with a worse postural stability in balance tests^72,73^. No such correlations were found in the present dataset. Also, vection strength has been described as positively correlated with the Romberg quotient^69^, but again these two parameters showed no correlation in the present experiment (see Table 1).

### Task performances unrelated to redirection thresholds

The tasks introduced under “Blind locomotion control and interoceptive awareness” (Fig. 2) produced results that were uncorrelated with participants’ redirection thresholds. In what way could the absence of a statistical relationship inform us about the nature of the investigated associations? The results provoke the assumption that an individual’s sensitivity to redirection manipulation barely depends on his or her tendency to veer to either side when attempting to walk blind on a straight path. This sensitivity is also unlikely to be a function of an individual’s interoceptive abilities.

In view of our tentative explanation with respect to the association between blind postural control and redirection thresholds (see above), it may seem puzzling that similar arguments would not also hold for keeping a straight path during blind walking. More particularly, once we assume that persons with an instable postural control would recognize even small amounts of artificially imposed gait perturbances especially fast, should we not also assume this for persons with an instable “straight-walking” tendency? Not necessarily. Veering as a dynamic process during locomotion is hardly comparable to balancing as an act of keeping a static body position. Accordingly, balance and veering measures were clearly uncorrelated in the present sample. Notably, a previous study has shown one parameter of veering to be associated with the ability to recognize an experimenter-induced curvature while attempting to walk straight ahead^35^. The authors did not find support for their original hypothesis of an association between spontaneous blind veering and the detection of forced path curvature during blind locomotion (in a 2AFC task; forced leftward or forced rightward). However, they incidentally found that the *variability* of direction from one step to the next during spontaneous, not redirected locomotion would predict accuracy of curvature detection (higher variability associated with worse detection). They concluded that this variability could form a source of vestibular or proprioceptive information that could be used to assess the presence or absence of non-self-generated gait deviations. We have analysed the relation between participants’ redirection thresholds and their step-direction variability in the veering task and found it far from significant. This non-replication of Kallie *et al*.^35^ may be explained by differences in the two procedures: In their experiment redirection was accomplished by a guiding handle during blind walking, while in the present experiment we used a rotation of the visual scenery on the HMD.

Interoceptive sensitivity, assessed with the heartbeat detection task, was examined as a further measure of non-visual body awareness. We assumed that a better monitoring of one’s internal bodily state would be associated with a better detection of being redirected. However, no significant correlation emerged between participants’ cardiac awareness and their redirection thresholds. A word of caution is needed about the suitability of the heartbeat detection task for the present context. Although, as a group, participants were able to distinguish between synchronous and asynchronous conditions, performance was not overwhelming (mean success rate: 54.7% against a chance rate of 50%). On an individual basis only 5 out of 56 participants (in four participants the interoception task could not be performed) achieved a performance statistically better than chance. A less difficult interoceptive task may have produced a different result. However, also individual scores on the somatosensory amplification scale reflecting sensitivity to bodily signals were uncorrelated to curvature detection in the VE. Together, these null findings in cardiac monitoring and questionnaire-based interoceptive sensitivity make it improbable that awareness of gait distortions heavily relies on interoceptive variables.

## Conclusions

The most prominent relation to redirection thresholds was found in participants’ adjustments of the subjective visual vertical. In fact, the performance in the rod-and-frame task was the only variable that remained a significant predictor of individual redirection thresholds in the multivariate analysis. This makes us conceive redirection manipulations in VR as a “visual capture of gait”. That is, VR users are made believe their eyes to the extent that their locomotor apparatus and vestibular system are duped. However, on top of this visual-dominance effect, some higher-order sensory integration processes may also influence a person’s sensitivity to notice an imposed alteration of gait direction. The associations we found between participants’ behaviour in the VR setting and the detection of self-motion and visually-assisted postural stability suggest that those, who achieve an especially rapid visual-vestibular integration are also those with particularly low redirection thresholds. It remains to be investigated how visually more sophisticated VEs than the one used in the present study influence the pattern of findings reported here.

## Data Availability

The datasets generated and/or analysed during the current study are available in the Open Science Framework repository (project title: “Visual capture of gait during redirected walking”; https://osf.io/5dmkb/).
